# Clinical implications of hypoxia biomarker expression in head and neck squamous cell carcinoma: a systematic review

**DOI:** 10.1002/cam4.460

**Published:** 2015-04-27

**Authors:** Justin E Swartz, Ajit J Pothen, Inge Stegeman, Stefan M Willems, Wilko Grolman

**Affiliations:** 1 Department of Otorhinolaryngology – Head and Neck Surgery, University Medical Center UtrechtUtrecht, The Netherlands; 2 Brain Center Rudolph Magnus, University Medical Center UtrechtUtrecht, The Netherlands; 3 Department of Pathology, University Medical Center UtrechtUtrecht, The Netherlands

**Keywords:** head and neck neoplasms, hypoxia, hypoxia-inducible factor 1, personalized medicine, tumor microenvironment

## Abstract

Awareness increases that the tumor biology influences treatment outcome and prognosis in cancer. Tumor hypoxia is thought to decrease sensitivity to radiotherapy and some forms of chemotherapy. Presence of hypoxia may be assessed by investigating expression of endogenous markers of hypoxia (EMH) using immunohistochemistry (IHC). In this systematic review we investigated the effect of EMH expression on local control and survival according to treatment modality in head and neck cancer (head and neck squamous cell carcinoma [HNSCC]). A search was performed in MEDLINE and EMBASE. Studies were eligible for inclusion that described EMH expression in relation to outcome in HNSCC patients. Quality was assessed using the Quality in Prognosis Studies (QUIPS) tool. Hazard ratios for locoregional control and survival were extracted. Forty studies of adequate quality were included. HIF-1a, HIF-2a, CA-IX, GLUT-1, and OPN were identified as the best described EMHs. With exception of HIF-2a, all EMHs were significantly related to adverse outcome in multiple studies, especially in studies where patients underwent single-modality treatment. Positive expression was often correlated with adverse clinical characteristics, including disease stage and differentiation grade. In summary, EMH expression was common in HNSCC patients and negatively influenced their prognosis. Future studies should investigate the effect of hypoxia-modified treatment schedules in patients with high In summary, EMH expression. These may include ARCON, treatment with nimorazole, or novel targeted therapies directed at hypoxic tissue. Also, the feasibility of surgical removal of the hypoxic tumor volume prior to radiotherapy should be investigated.

## Introduction

Despite improvement of surgical and radiotherapeutic techniques, as well as the introduction of systemic therapies including cisplatin or cetuximab, 5-year survival rates for patients with head and neck squamous cell carcinoma (HNSCC) remain low 1. Currently, staging and treatment selection is based only on clinical staging using the AJCC TNM-classification. However, awareness increases that not all squamous cell carcinomas are the same, but have different tumor biology 2. These differences could have an even greater impact on treatment outcome than mere clinical staging. An example is infection with the human papillomavirus (HPV) in oropharyngeal squamous cell carcinoma (OPSCC): HPV-associated (HPV+) OPSCC cancers show a much better response to radio- and chemotherapy than non-HPV-associated (HPV−) OPSCC 3,4. In this line there is a clear need for other novel biomarkers to predict sensitivity to a particular treatment modality, or to identify which patients might benefit from adjuvant therapies.

One possible target or prognosticator is tumor hypoxia. Hypoxia is defined as a mismatch between cellular oxygen demand and supply. The causes of hypoxia can roughly be divided into two categories: acute or chronic hypoxia. Acute, or perfusion-limited, hypoxia, occurs when there is insufficient oxygen supply to cells due to compromise of the supplying blood vessels. Acute hypoxia causes electrolyte imbalances and an increase in intracellular hydrogen sulfide. When this occurs in specialized hypoxia-sensing cells, such as glomus or smooth muscle cells, this leads to a systemic response, such as vasodilation 5. In contrast, chronic hypoxia triggers a cellular response in individual cells only after several hours of hypoxia 6. Chronic hypoxia is often caused by diffusion-limitations which occur when the distance from a cell to the nearest blood vessel is too large for adequate cellular oxygenation 7. Because of expansive tumor growth, chronic hypoxia often occurs in solid tumors, including HNSCC 8. HNSCC is often treated with radiotherapy which depends on oxygen for free radical formation to induce DNA strand breaks and cell death. Because of the need for oxygen, tumor hypoxia causes decreased sensitivity to radiotherapy. Separately, hypoxia is thought to induce tumor progression and a more aggressive phenotype 9. Therefore, the hypoxic status of a tumor could possibly contribute in identifying the treatment option that offers the best prognosis to an individual patient. For example, surgical removal of the hypoxic component before radiotherapy might be preferable above radiotherapy alone, when reduced sensitivity to primary radiotherapy is expected. Alternatively, hypoxia-sensitizing radiotherapy schedules such as accelerated radiotherapy with carbogen-breathing and nicotinamide (ARCON) or addition of a hypoxia-sensitizing drug like nimorazole may be considered 10,11.

Several ways of assessing tumor hypoxia have been described 12. This includes the invasive Eppendorf *p*O_2_ histography, that uses polarographic needles to measure tissue *p*O_2_ in vivo. Tissue biomarkers for hypoxia may also be used to assess tumor hypoxia histologically. The use of exogenous biomarkers, for instance of the nitroimidazole class, is also invasive, as they have to be administered to patients intravenously before excision of the tissue. Finally, various endogenous biomarkers for hypoxia (EMHs) exist, that can be used to assess the hypoxic state using IHC, with no need for additional invasive procedures other than routine diagnostic biopsy. The most important endogenous biomarkers are part of the hypoxia-inducible factor 1(HIF)-1 pathway. HIF-1 is upregulated under hypoxia to improve cellular survival in a hypoxic microenvironment. This basic helix-loop-helix transcription factor consists of an alpha (HIF-1a) and beta (HIF-1b or ARNT) subunit. Both are constitutively expressed, but under normoxic conditions HIF-1a is quickly degraded by hydroxylation and binding to the VHL protein 13,14. In the hypoxic state, hydroxylation of HIF-1a is inhibited, causing stabilization, enabling interaction with HIF-1b and increased transcription of its downstream targets. Another EMH is osteopontin (OPN), which is expressed independently of HIF-1a and is involved in the adhesive cell–matrix interaction and is considered a protein involved in tumor development and progression 15,16. A brief review of the studied EMHs is shown in Box Box 1.

**Box 1 Endogenous markers of hypoxia**

**Table:** 

Biomarker	Role
HIF-1a	HIF-1alpha is the alpha subunit of the HIF-1 transcription factor, which is part of the cellular defense mechanisms to survive in a hypoxic state. Under normoxic conditions it is quickly degraded by prolyl hydroxylase (PHD) 1–3. Under hypoxic conditions, PHD activity is inhibited, causing overexpression of HIF-1a. As a transcription factor, HIF-1 stabilization causes increased transcription of its downstream targets through hypoxia-responsive elements (HRE) in the DNA
HIF-2a	HIF-2alpha is also a transcription factor in the HIF family, but has distinct other downstream targets. HIF-2a stabilization under hypoxia occurs through the same mechanism as HIF-1a
CA-IX	As hypoxic cells rely on anaerobic metabolism, intracellular pH will drop because of lactate formation. Carbonic anhydrase (CA) IX is a downstream target of HIF-1 involved in pH regulation 85.
GLUT-1	A downstream target of HIF-1a. In hypoxic conditions, additional glucose is required for the anaerobic metabolism. There are many members in the glucose transporter (GLUT-) family, but GLUT-1 is specifically upregulated by HIF-1a
OPN	Osteopontin (OPN) is an integrin-binding protein of the SIBLING family (small integrin-binding ligand N-linked glycoprotein) and was first discovered in bone tissue. It promotes cellular survival through the NF-*κβ* pathway by reducing cell peroxide levels 86. It is upregulated independent of HIF-1a under hypoxia

HIF-1, hypoxia-inducible factor 1.

Several (narrative) reviews are currently available on the effect of HIF-1a expression on local control and survival in patients with HNSCC. However, to our knowledge, no systematic reviews have studied EMH expression from a clinical approach, by systematically comparing the effect of all EMHs according to treatment outcome and taking into account differences between subsites. In the present study, we investigate which biomarkers are used to determine tumor hypoxia in HNSCC, as well as the effect of overexpression on clinical outcome.

## Methods

### Search strategy

**Figure 1:**
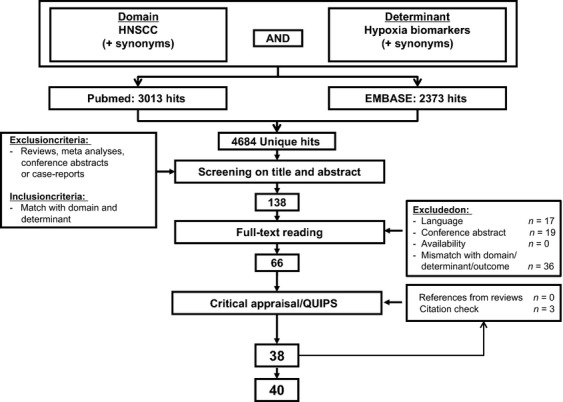
Study selection process. Study selection flowchart. Of the 66 suitable papers, 38 were found of adequate quality. A citation check yielded three additional results, of which two were of adequate quality. In total, 40 studies were included.

A systematic review was performed in PubMed/MEDLINE and EMBASE. The search strategy is shown in Table S1. Briefly, a search was performed for studies that described the domain (“HNSCC”) and the determinant (“hypoxia”/EMHs) or synonyms of these terms in the title or abstract or as MeSH terms. The MEDLINE GENE database was used to identify synonyms of the various EMHs. Abstracts were screened based on predetermined in- and exclusion criteria by two authors independently (Fig.1). Full-text analysis of potentially relevant abstracts was performed and a final selection was made. At all stages, differences were resolved by discussion. The review was limited to EMHs that were studied in more than two articles.

Relevant full text papers were appraised for risk of bias using the Quality in Prognosis Studies (QUIPS) tool, that has been developed for systematic appraisal in studies of prognostic factors 17. Using QUIPS, a risk of bias is determined, based on the study design and the reported results. For each of the six domains within QUIPS, the risk of bias was judged low (0 points), moderate (1 point) or high (2 points), based on three to seven predefined criteria per domain. For the current review, the following criteria were used: the source population should consist of a consecutive cohort of patients. Baseline characteristics should include T- and N-staging, as well as the treatment modality. Studies disclosing loss to follow-up and that confirmed whether censored patients were known to be alive at the moment of analysis were valued highest in the “study attrition” appraisal. In the correction for confounding appraisal, studies that investigated potential confounding effects of T- and N-staging, as well as treatment modality were valued highest. Finally, studies that scored a low risk of bias (≤3) were included.

### Data extraction and meta-analysis

Extracted data included number of patients, disease stage, tumor subsite, treatment, biomarkers, and corresponding cutoffs and outcome. The studied outcomes were the hazard ratios (HR) for locoregional control (LRC), overall survival (OS), disease-free survival (DFS) and disease-specific survival (DSS). If a HR was not described, but a Kaplan–Meier curve was available, the curve was digitized using the open-source Engauge Digitizer software (http://digitizer.sourceforce.net) and a univariate HR was estimated through the methods of Tierney et al. 18. Meta-analysis was considered only if studies used the same cutoff values for EMH positivity and described patient cohorts were comparable in terms of treatment and disease stage. A review protocol was not previously published. Results are presented in accordance with the PRISMA statement for systematic reviews 19.

## Results

### Study selection

The search in EMBASE and PubMed yielded 4684 unique publications. Abstract screening yielded 138 potentially interesting papers, of which the full text was requested. Of these papers, 17 were not in English, Dutch or German, 19 were conference abstracts with no full text paper available and 36 were excluded for various reasons of mismatch with the domain, determinant, or outcome. Sixty-six papers remained for critical appraisal. Relevant reviews were read, references were screened and a citation check was performed using Web of Science. This yielded three additional papers. The study selection process is shown in Figure1.

## Critical appraisal

Using the QUIPS criteria, the 66 and 3 papers identified through the search and citation checks, respectively, were appraised (Table1 for included studies, Table S2 for excluded studies). In many papers it was not described whether the cohort was consecutive. Also, loss to follow-up and the characteristics of patients lost to follow-up were rarely reported, resulting in a high risk of bias in the “Study Attrition” domain. Many studies used data-dependent cutoffs for their prognostic factor assessment, scoring lower on the “Prognostic factor” domain. Also, the treatment modality was often not included in multivariate analysis. As the effect of hypoxia might be different for each treatment modality (e.g., surgery, radiotherapy, or chemoradiation [CRT]), these papers scored a higher risk of bias in the “Confounding” domain, except when the cohort received uniform treatment. A score was calculated as described before, however, the study attrition score was omitted in this final risk of bias score. Forty papers remained for final analysis.

**Table 1:** Critical appraisal of included studies and description of biomarkers

Study	SP	SA	PF	O	C	AR	B	HIF-1a	HIF-2a	CA-IX	GLUT-1	OPN
Aebersold et al. 20	L	H	L	L	M	L	1	•				
Avirović et al. 35	M	L	M	L	M	L	3					•
Brockton et al. 48	L	H	M	L	M	L	2			•		
Brockton et al. 33	M	H	M	L	M	L	3			•	•	
Cabanillas et al. 53	H	H	L	L	M	L	3	•				
Chien et al. 36	H	H	L	L	L	M	3					•
Chien et al. 37	M	L	L	L	M	L	2					•
Choi et al. 57	M	H	L	L	M	M	3	•				
Choi et al. 38	M	L	L	L	M	M	3			•		
Dos Santos et al. 39	M	L	L	L	L	M	2	•				
Douglas et al. 22	L	H	L	L	L	L	0	•		•		
Dunkel et al. 40	L	H	L	L	M	M	2	•				
Eckert et al. 41	H	L	L	L	M	L	3	•		•		
Eckert et al. 42	H	L	L	L	L	L	2	•			•	
Eriksen et al. 61	L	L	L	L	L	L	0			•		
Fillies et al. 49	H	H	M	L	L	L	3	•				
Grimm et al. 44	H	L	L	L	M	L	3				•	
Hong et al. 56	L	L	L	L	L	L	0	•				
Hui et al. 31	L	H	L	L	L	L	0	•		•		
Jonathan et a. 30	M	L	L	L	L	L	1			•	•	
Kim et al. 51	H	L	M	L	L	L	3			•		
Kitagawa et al. 32	H	L	M	L	L	L	3	•				
Kwon et al. 23	H	L	L	L	M	L	3	•		•	•	
Le et al. 58	H	L	M	L	L	L	3			•		•
Liang et al. 45	L	H	L	L	H	L	2	•	•			
Nordsmark et al. 29	M	L	L	L	M	M	3	•		•		•
Pérez-Sayáns et al. 55	M	L	L	L	M	M	3			•		
Rademakers et al. 24	M	L	M	L	L	L	2			•		
Rahimi et al. 60	L	H	L	L	L	L	0	•		•		
Roh et al. 52	L	L	M	L	M	L	2	•		•	•	
Schrijvers et al. 25	L	L	M	L	L	L	1	•		•	•	
Silva et al. 21	M	H	L	L	M	L	2	•				
Van den Broek (2009)	L	H	M	L	L	L	1	•		•		
Wachters et al. 26	L	H	L	L	L	L	0	•		•		•
Wan et al. 59	M	H	M	L	L	L	2	•				
Wildeman et al. 27	M	L	L	L	M	L	2	•		•		
Winter et al. 54	M	L	L	L	M	L	2	•	•	•		
Xueguan et al. 28	L	L	L	L	M	M	2	•				
Zheng et al. 46	M	H	L	L	M	M	3	•				
Zhu et al. 47	L	H	M	L	L	L	1	•	•			
Totals								27	3	21	7	6

Low, 0; Moderate, 1; High, 2 points. SA was not included. Studies with a bias score >3 were excluded. Appraisal of these studies is described in supplementary table S2. SP, study participation; SA, study attrition; PF, prognostic factor; O, Outcome; C, Confounding; AR, statistical analysis and reporting. B, Bias score according to QUIPS; HIF-1, hypoxia-inducible factor 1. The EMHs that were described in each study are shown with a filled dot (*example of filled dot*).

## Endogenous markers of hypoxia

The included studies described the effect of EMH expression across all subsites in the head and neck area. However, many studies analyzed different staining patterns, methods, and cutoff values to define EMH positivity. For instance, some studies used an H-score that combined staining proportion and intensity, while others only scored staining proportion or intensity. Because of this heterogeneity, it was deemed that data pooling and subsequent meta-analysis were not appropriate, as this might introduce bias.

**Figure 2:**
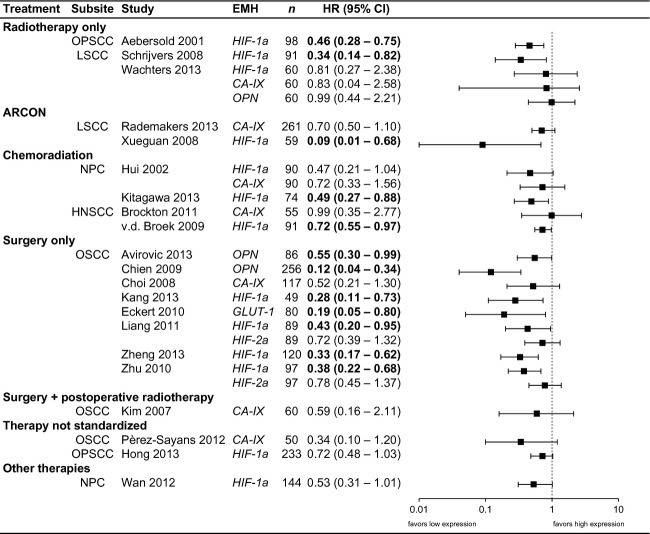
Forest plot: Overall survival and EMH Expression. Visual summary of studies that described overall survival. ARCON, accelerated radiotherapy, carbogen gas, and nicotinamide. HRs < 1 indicate beneficial prognosis for nonhypoxic tumors. Therapy not standardized: All treatment modalities were analyzed in a single cohort and results were not presented according to therapy. The studies of Pérez-Sayáns and Hong describe their entire cohort of patients, receiving any treatment. In the study of Wan, patients were randomized between neoadjuvant radiotherapy or chemoradiation, followed by concurrent chemoradiation. EMH, endogenous markers of hypoxia.

EMH expression was often correlated with adverse clinical parameters, such as T-stage, N-stage, or differentiation grade, as shown in Tables2 through 7. The effect of EMH expression is discussed per treatment strategy, as hypoxia may influence outcome of various treatment strategies differently. As a summary, a forest plot for OS across all treatment strategies is shown in Figure2.

## Primary radiotherapy or ARCON

Eleven studies were identified that studied the clinical effect of EMHs in patients treated with radiotherapy (XRT) or the hypoxia-sensitizing treatment of accelerated radiotherapy, in combination with carbogen gas breathing and intravenously administered nicotinamide (ARCON) 20–30. Results are summarized in Table2. Most studies identified a worse outcome in patients with high EMH expression. This finding appears to be present across all subsites within the head and neck region, including the oropharynx 20,21, the larynx 23,25, and the nasopharynx 28, as well as in a study that analyzed patients with cancers of several subsites 29. In this last study multiple biomarkers of hypoxia were studied. HIF-1a expression predicted significantly worse LRC. While LRC was lower in patients with high expression of CA-IX and OPN, this did not reach statistical significance.

**Table 2:** Clinical outcome: radiotherapy/ARCON

Study	Treatment	Stage	EMH	Pos/*n*	Cutoff	Correlations	LRC	OS	DFS	DSS
Oropharyngeal carcinoma										
Aebersold et al. 20	XRT	Any	HIF-1a	92/98	10% N	Tumor grade		**0.46 (0.28–0.75)**	**0.50 (0.30–0.83)**	
Silva et al. 21	XRT		HIF-1a	43/79	10%	Low Hb	**0.2 (0.1–0.42)**			
Laryngeal carcinoma										
Douglas et al. 22	XRT	I–II	HIF-1a	124/271	10% N	None	0.96 (0.79–1.16)			LR *P* = 0.23
Kwon et al. 23	XRT	I–II	HIF-1a	7/42	50% N	ns	**0.13 (0.02–0.82)**			
		I–II	CA-IX	17/42	30% M	ns	**0.11 (0.01–0.96)**			
Rademakers et al. 24	ARCON/XRT^1^	III–IV	CA-IX	132/261	Med^2^	None		0.7 (0.5–1.1)		
Schrijvers et al. 25,3	XRT	I–II	HIF-1a	46/91	0.5% N	None		**0.34 (0.14–0.82)**		
		I–II	CA-IX	39/91	12.5% M	None	**0.34 (0.14–0.85)**	ns		
		I–II	GLUT-1	53/91	35% M	ns	ns			
Wachters et al. 26	XRT	I–II	HIF-1a	47/60	0.5% N	None	0.93 (0.26–3.45)	0.81 (0.27–2.38)		
		I–II	CA-IX	11/60	12.5% M	None		0.83 (0.04–2.58)		
		I–II	OPN	20/60	0.5% C	ns		0.99 (0.44–2.21)		
Wildeman et al. 27	XRT	Any	HIF-1a	59	N/M %^4^	ns	1.08 (0.91–1.29)^5^			
		Any	HIF-1a	59	Int	ns	0.92 (0.56–1.49)^5^			
		Any	CA-IX	59	int	ns	1.21 (0.96–1.52)^5^			
Nasopharyngeal carcinoma										
Xueguan et al. 28	ARCON	Any	HIF-1a	40/59	10% N	None	0.41 (0.06–2.69)	**0.09 (0.01–0.68)**^6^	**0.26 (0.07–0.97)**	
Multiple subsites										
Nordsmark et al. 29	XRT	Any	HIF-1a	19/59^7^	50% N	ns	**0.22 (0.06–0.81)**			
			CA-IX	26/57^7^	10% M	ns	0.35 (0.12–1.01)			
			OPN	17/57	Int D	ns	0.83 (0.35–2.00)			
Jonathan et al. 30	ARCON	Any	CA-IX	29/58	25% M	ns	**4.23 (1.07–16.76)**^6^	ns		
			GLUT-1	29/58	Int D	ns	ns	**LR** ***P***** = 0.001**		

The outcomes locoregional control (LRC), overall survival (OS), disease-free survival (DFS), and disease-specific survival (DSS) are shown as hazard ratio (95% confidence interval). Hazard ratios <1 indicate beneficial prognosis for nonhypoxic tumors. Significant values are shown in bold Cutoff: EMHs were scored according to nuclear (N), membranous (M), cytoplasmic (C), or diffuse (D) staining patterns. XRT: radiotherapy. ARCON; accelerated radiotherapy, carbogen gas breathing and nicotinamide. Pos: number of patients with staining above the mentioned cutoff. LR: Logrank test. ns: not specified. Multiple subsites, patients were not analyzed per subsite. EMH, endogenous markers of hypoxia; HIF-1, hypoxia-inducible factor 1.

1 Patients were randomized between ARCON and XRT.

2 Computerized image analysis was performed and the median value was used in statistical analyses.

3 Supraglottic carcinomas only.

4 Analyses were performed using the proportion of membranous or nuclear staining cells as a continuous variable, no cutoff was used. Number of positive staining patients is therefore not relevant.

5 Presented numbers are odds-ratios for 2-year locoregional recurrence, not hazard ratios.

6 Last surviving patient was scored as deceased to enable HR-calculation, because of 100% survival in one arm.

7 Data on immunohistochemical analysis were available for 59 of 67 patients (HIF-1a) and 57/67 patients (CA-IX).

The study of Rademakers et al. 24 of 261 patients randomized between treatment with XRT or ARCON did not find better OS in low CA-IX expressing laryngeal cancer patients (LSCC). Unfortunately, no separate data were presented for XRT and ARCON. The authors did report differences in OS between different staining patterns: a perinecrotic staining pattern, in which cells stain more strongly as the distance to the nearest blood vessel increases, was associated with worse OS (*P* < 0.01) and LRC (*P* = 0.01) when compared to diffuse or no expression of CA-IX. Surprisingly, in the study of Jonathan et al. 30 of 58 HNSCC patients treated only with ARCON, a better outcome was observed in patients with high EMH expression. In this small study, CA-IX expression was mostly low. Using a cutoff at the median value of expression, the authors describe no significant correlation with the outcome. Finally, the 80th percentile (25% membranous expression) was used as a cutoff value that found a significantly better outcome for patients with high CA-IX levels.

## Primary CRT

Only four studies were available that studied EMH expression in a cohort of patients treated with CRT only 31–34. A significant effect of EMH expression on survival was found in two 32,34. Kitagawa et al. 32 described a cohort of 74 nasopharyngeal cancer (NPC) patients treated with CRT (with exempt of seven patients that did not receive chemotherapy because of kidney failure), patients with more than 10% HIF-1a expression had significantly worse OS. Van den Broek et al. studied HIF-1a expression in a cohort of 91 HNSCC patients and described worse OS, but not LRC in patients with higher HIF-1a expression 34. Hui et al. studied a cohort of 90 NPC patients and found a trend toward better outcome in low HIF-1a-expressing patients, but did not find a similar trend for CA-IX 31. Brockton et al. studied CA-IX and GLUT-1 expression in a smaller cohort of 58 patients with primary tumors from various subsites in the head and neck and also did not find a correlation with survival 33 Results are summarized in Table3.

**Table 3:** Clinical outcome: primary chemoradiation

Study	Stage	EMH	Pos/*n*	Cutoff	Correlations	LRC	OS
Nasopharyngeal cancer							
Hui et al. 31	III–IV	HIF-1a	32/90	5% N	None		0.47 (0.21–1.04)
		CA-IX	32/90	5% M	None		0.72 (0.33–1.56)
Kitagawa et al. 32	Any	HIF-1a	27/74^1^	10% N	None		**0.49 (0.27–0.88)**
Multiple subsites							
Brockton et al. 33	II–IV	CA-IX	23/46^2^	Med^3^	None		0.99 (0.35–2.77)
		GLUT-1	24/47^2^	Med	None		LR *P* = 0.79
Van den Broek et al. 34	Any	HIF-1a	91	N/M^4^	ns	0.64 (0.36–1.12)	**0.72 (0.55–0.97)**
		CA-IX	91	M/C^4^	ns	0.73 (0.43–1.23)	ns

The outcomes locoregional control (LRC) and overall survival (OS) are shown as hazard ratio (95% confidence interval). None of the studies reported disease free or disease-specific survival. Hazard ratios <1 indicate beneficial prognosis for nonhypoxic tumors. Significant values are shown in bold. Cutoff: EMHs were scored according to nuclear (N), membranous (M), cytoplasmic (C), or diffuse (D) staining patterns. Pos: number of patients with staining above the mentioned cutoff. LR: Logrank test. ns: not specified. Multiple subsites: patients were not analyzed per subsite. EMH, endogenous markers of hypoxia; HIF-1, hypoxia-inducible factor 1.

1 Seven of 74 patients received radiotherapy only due to kidney failure.

2 Total 55 patients, data were missing because of missing or folded TMA cores.

3 Computerized image analysis was performed, staining pattern was not taken into account.

4 Nuclear or membranous expression was analyzed as a continuous variable.

## Primary surgery

Thirteen studies studied EMH expression in patients treated with primary surgery only 35–47. All studies concerned oral cavity carcinoma (OSCC) and all studies, except two, described that in surgically treated patients, HIF-1a expression significantly decreased the prognosis. Choi et al. 38 investigated CA-IX expression in a cohort of 118 patients and did not find an association with prognosis. The study of Dos Santos et al. 39 describes a small subgroup of 36 patients treated only with surgery in a larger cohort of 66 OSCC patients and did not find a difference between high and low HIF-1a-expressing patients. Results are summarized in Table4.

**Table 4:** Clinical outcome: primary surgery

Study	Stage	EMH	Pos/*n*	Cutoff	Correlations		OS	DFS	DSS
Oral cavity									
Avirović et al. 35	Any	OPN	48/86	71% C	N-stage, disease stage		**0.55 (0.3–0.99)**		
Chien et al. 36	Any	OPN	30/94	10% C	T-stage, N-stage, tumor thickness, tumor necrosis			**LR** ***P***** < 0.001**	
Chien et al. 37,1	Any	OPN	192/256^6^	10% C	T-stage, N-stage, disease stage		**0.12 (0.04–0.34)**		
Choi et al. 38	Any	CA-IX	64/117	5% M	None		0.52 (0.21–1.30)		
Dos Santos et al. 39	Any	HIF-1a	15/36	6/9^2^	None				LR *P* = 0.7
Dunkel et al. 40	I	HIF-1a	16/44	Int	ns			**LR** ***P***** = 0.022**	LR *P* = 0.29
Kang et al. 43,3	I–III	HIF-1a	43/49	10%^3^	T-stage, N-stage, tumor grade		**0.28 (0.11–0.73)**	**0.34 (0.15–0.79)**	
Eckert et al. 41	Any	HIF-1a	80^4^	3–4 vs. 6–8 C^5^	T-stage		**0.21 (0.06–0.72)**		
				3–4 vs. 9–12 C^5^			**0.19 (0.05–0.8)**		
Eckert et al. 42	Any	GLUT–1	80^6^	0–2 vs. 3–4 M^5^	None				0.71 (0.2–2.54)
				0–2 vs. 6–8 M^5^					**0.29 (0.12–0.71)**
				0–2 vs. 9–12 M^5^					0.5 (0.15–1.63)
Grimm et al. 44	Any	GLUT–1	161	50% M/C^7^	ns				**0.58 (0.37–0.91)**
Liang et al. 45	Any	HIF-1a	89	25% N/C^7^	Tumor grade, N-stage		**0.43 (0.20–0.95)**		
		HIF-2a	89	25% N/C^7^	T-stage		0.72 (0.39–1.32)		
Zheng et al. 46	Any	HIF-1a	120	1% N	N-stage, disease stage		**0.33 (0.17–0.62)**	**0.30 (0.16–0.75)**	
Zhu et al. 47	Any	HIF-1a	z97	1% N	T-stage, N-stage, tumor grade		**0.38 (0.22–0.68)**	**0.44 (0.26–0.75)**	
		HIF-2a	97^4^	1% N	T-stage, microvessel density		0.78 (0.45–1.37)	0.87 (0.51–1.47)	

The outcomes overall survival (OS), disease free survival (DFS) and disease specific survival (DFS) are shown as hazard ratio (95% confidence interval). None of the studies reported locoregional control. Hazard ratios <1 indicate beneficial prognosis for nonhypoxic tumors. Significant values are shown in bold. Cutoff: EMHs were scored according to nuclear (N), membranous (M), cytoplasmic (C), or diffuse (D) staining patterns. Pos: number of patients with staining above the mentioned cutoff. LR: Logrank test. ns: not specified. Multiple subsites: patients were not analyzed per subsite. EMH, endogenous markers of hypoxia; HIF-1, hypoxia-inducible factor 1.

1 Patients from Chien et al. 36 were also included in the sample from Chien et al. 37.

2 A score range 0–9 was calculated based on staining proportion and intensity. Staining pattern was not disclosed.

3 The scored staining pattern was not disclosed.

4 Negative expression (score 0–2): 11, weak (3, 4): 24, moderate (6–8) 38, strong (9–12): 7 patients.

5 A score range 0–12 was calculated based on staining proportion and intensity.

6 Negative expression (score 0–2): 32, weak (3, 4): 13, moderate (6–8) 21, strong (9–12): 11 patients.

7 Both membranous and cytoplasmic (M/C) or nuclear and cytoplasmic (N/C) staining cells were scored positive.

## Surgery and postoperative radiotherapy

Eight studies were available that studied EMH expression in a cohort of patients treated with surgery and postoperative radiotherapy 39,48–54. Surprisingly, two studies described better prognosis in high HIF-1a expressing patients 39,49. The study of Fillies et al. 49 described the effect of HIF-1a expression on clinical outcome and found that HIF-1a expression above 5% results in better survival in OSCC patients. The study of Dos Santos et al. describes a small subgroup of 30 patients treated with surgery and postoperative radiotherapy within a study of 66 OSCC patients 39. The study of Winter et al. 54 describe significantly worse outcome for HNSCC patients with high HIF-1a expression. All other studies did not show a difference in outcome when patients were stratified according to EMH expression. Results are summarized in Table5.

**Table 5:** Clinical outcome: primary surgery + postoperative radiotherapy

Study	Stage	EMH	Pos/n	Cutoff	Correlations	Favor	LRC	OS	DFS	DSS
Oral cavity										
Brockton et al. 48	Any	CA-IX	17/61	p75^1^	ns	L				0.26 (0.06–1.05)
Dos Santos et al. 39	Any	HIF-1a	16/30^2^			H				**3.41 (1.13–10.34)**
Fillies et al. 49	I–II	HIF-1a	45/85	5% N	None	H		**LR** ***P***** < 0.05**^3^	**LR** ***P***** = 0.02**	
Han et al 50	II	HIF-1a	4/33	10% N	ns	L				
Kim et al. 51	Any	CA-IX	38/60	10% C/M	Tumor grade, subsite, smoking	L		0.59 (0.16–2.11)	0.85 (0.33–2.23)	
Roh et al. 52	II	HIF-1a	6/43	1% N	None	L	LR *P* = 0.154			0.37 (0.13–1.03)
		CA-IX	26/43	10% M	Tumor thickness	L	LR *P* = 0.857			LR *P* = 0.159
		GLUT-1	31/43	50% M	Tumor thickness, n-stage	L	LR *P* = 0.416			LR *P* = 0.060
Laryngeal cancer										
Cabanillas et al. 53	Any	HIF-1a	75/106	10% N	T-stage, disease stage	N		LR *P* = 0.8		LR *P* = 0.5
Multiple subsites										
Winter et al. 54	Any	HIF-1a	45/151	medN^3^	Disease stage	L		LR *P* = 0.08	**LR** ***P***** = 0.02**	**LR** ***P***** = 0.02**
		HIF-2a	21/151	Med N/C^3^	None	L		LR *P* = 0.43	LR *P* = 0.10	LR *P* = 0.16
		CA-IX	92/151	Med M^3^	ns	–		LR *P* = 0.3	LR *P* = 0.2	LR *P* = 0.1

The outcomes locoregional control (LRC), overall survival (OS), disease-free survival (DFS), and disease-specific survival (DSS) are shown as hazard ratio (95% confidence interval). Hazard ratios <1 indicate beneficial prognosis for nonhypoxic tumors. Significant values are shown in bold. Cutoff: EMHs were scored according to nuclear (N), membranous (M), cytoplasmic (C), or diffuse (D) staining patterns. Pos: number of patients with staining above the mentioned cutoff. Favor: L, better outcome for low expression; H, better outcome for high expression; N, no difference between arms. LR: Logrank test. ns: not specified. Multiple subsites: patients were not analyzed per subsite. EMH, endogenous markers of hypoxia; HIF-1, hypoxia-inducible factor 1.

1 Computerized image analysis, scoring pattern and value not disclosed.

2 Subgroup within a larger study of 66 patients.

3 Exact value not disclosed.

## Other treatment strategies

Seven studies describe data from larger cohorts treated with various treatment modalities, depending on localization and staging 55–61. Only the studies of Rahimi and Le identified a significant correlation between EMH expression and outcome. Rahimi found significant better DFS and improved, but not significantly different LRC for patients expressing no or very low levels (<1%) of HIF-1a 60. Similar results were not obtained for CA-IX. Le et al. 58 studied both CA-IX and OPN expression and found better survival in patients with low CA-IX expression. Pérez-Sayáns et al. (CA-IX), Hong (HIF-1a), and Choi (HIF-1a) did not find a correlation with outcome 55–57. Wan et al. 59 studied 144 NPC patients randomized to receive neoadjuvant radiotherapy and neoadjuvant CRT followed by CRT and found better, although not statistically significant, OS in low HIF-1a expressing patients. The same results were obtained when both treatment arms were analyzed separately. Results are summarized in Table6.

**Table 6:** Clinical outcome: other treatment strategies

Study	Treatment	Stage	EMH	Pos/*n*	Cutoff	Correlations	LRC	OS	DFS	DSS
Oral cavity cancer										
Pérez-Sayáns et al. 55	Any	Any	CA-IX	23/50^1^	50% M	Disease stage		0.34 (0.1–1.2)^2^		
Oropharyngeal cancer										
Hong et al. 56	Any	Any	HIF-1a	137/233	10% N	T-stage, tumor grade		0.72 (0.48–1.03)		0.75 (0.46–1.22)
Rahimi et al. 60	XRT/CRT^3^	Any	HIF-1a	26/58	1% N		0.76 (0.55–1.01)		**0.81 (0.67–0.99)**	
			HIF-1a	NS/58	1% C		1.10 (0.72–1.67)		1.06 (0.83–1.35)	
		Any	CA-IX	NS/57	1% C		1.52 (0.71–3.23)		1.03 (0.81–1.32)	
			CA-IX	NS/57	1% M		0.93 (0.77–1.12)		1.01 (0.88–1.15)	
Wan et al. 59	nC+R/nC+CRT^4^	Any	HIF-1a	66/144^1^	5/16 C/N^5^			0.53 (0.31–1.01)		
Multiple subsites										
Choi et al. 57	Any	Any	HIF-1a	25/76	1% C	None		LR *P* = 0.237	0.55 (0.33–1.15)	
Eriksen et al. 61	6	Any	CA-IX	370	M^7^	None	LR *P* = 0.8			
Le et al. 58	Any	Any	CA-IX	29/94^8^	Int C^9^			**LR** ***P***** = 0.011**		**LR** ***P***** = 0.030**
		Any	OPN	70/84	Int D			NS		

The outcomes locoregional control (LRC), overall survival (OS), disease-free survival (DFS), and disease-specific survival (DSS) are shown as hazard ratio (95% confidence interval). Hazard ratios <1 indicate beneficial prognosis for nonhypoxic tumors. Significant values are shown in bold. Cutoff: EMHs were scored according to nuclear (N), membranous (M), cytoplasmic (C) or diffuse (D) staining patterns. Pos: number of patients with staining above the mentioned cutoff. LR: Logrank test. ns: not specified. Multiple subsites: patients were not analyzed per subsite. EMH, endogenous markers of hypoxia; HIF-1, hypoxia-inducible factor 1.

1 Twenty-three patients had intense staining, 18 patients had moderate staining and 9 patients had no staining.

2 Strong vs. no CA-IX staining.

3 Chemotherapy was added in the case of T4 or N3 disease.

4 Patients participated in a RCT between neoadjuvant chemotherapy followed by either radiotherapy or chemoradiation

5 A score was calculated from 0–16, based on staining proportion and intensity. Both cytoplasmic and nuclear patterns were scored.

6 Patients were randomized between radiotherapy or radiotherapy and the radiosensitizer nimorazole

7 Patients were analyzed in groups: <1%, 1–10%, 10–30%, and above 30%. None of these subgroups showed significantly better improvement compared to the other groups

8 Results for CA-IX and OPN were available for 94 and 84 patients, respectively, because of TMA core availability.

9 Expression was scored as negative, weak or strong by a single pathologist.

## Discussion

In this systematic review, we investigated expression of biomarkers for tumor hypoxia in relation to clinical outcome and treatment strategy. We identified 40 high-quality studies. EMH expression was common and associated to worse survival or LRC in most studies, although statistical significance was not always reached. In addition, EMH expression was often correlated with worse clinicopathological characteristics. Surprisingly, three studies found EMH expression to be associated to better outcome, but these mostly had a small sample size 30,39,49. Moreover, in studies that investigated multiple EMHs, high HIF-1a expression was often associated with worse outcome, while this was not always true for the other EMHs.

Chronic hypoxia is an important and highly prevalent problem in solid tumors 9. We observed that several adverse clinical parameters were often associated with higher EMH expression, such as the presence of cervical lymph node metastasis, higher T-stages, and worse differentiation grade. The latter two correlations support the hypothesis that hypoxia occurs more often in larger and faster growing tumors. Despite the correlations to clinical parameters, the presence of hypoxia was often an independent predictor of adverse outcome. One explanation might be that in the hypoxic microenvironment, several mechanisms are activated that improve cellular survival under these adverse circumstances. As the HIF-1a transcription factor is stabilized, transcription of proteins increases, including those involved in pH regulation (CA-IX), cellular metabolism (GLUT-1), but also genes involved in angiogenesis or oxygen transport. OPN expression occurs through a hypoxia-dependent, HIF-independent pathway and reduces cell death and apoptosis in hypoxic or reoxygenated cells, it may therefore signify a more aggressive tumor phenotype 15,16,62,63.

Radiotherapy relies on the formation of free oxygen radicals to induce DNA strand breaks and cell death 64. Also, radiotherapy causes apoptosis through stabilization of p53. The HIF-1 pathway upregulates proteins involved in epithelial-to-mesenchymal transition (EMT), including the transcription factor Snail 65,66. Radiation-induced DNA damage is reduced by EMT by emergence of cancer stem cells that express high levels of free radical-scavenging proteins 67. Moreover, Snail causes radioresistance by suppressing p53-mediated apoptosis 64,67. Snail also contributes to cisplatin resistance, which is often concurrently administered to patients as a radiosensitizer 68. Thus, hypoxia does not only directly affect (chemo-)radiosensitivity, but also indirectly through EMT.

In this review we identified several studies that show that increased EMH expression leads to worse LRC and survival. However, in patients that were surgically treated only, EMH expression was also associated with worse outcome 35–37,43,45. This supports the hypothesis that hypoxia also contributes to a more aggressive tumor phenotype as described above. Surprisingly, only few studies on HNSCC patients treated with surgery and postoperative (chemo)radiotherapy found an association between EMH expression and survival. A possible explanation is that decreased tumor volume, will lead to better sensitivity to radiotherapy, as was shown by Pameijer and Chen 69,70. Dunst and colleagues even described that the hypoxic tumor volume is a better prognosticator than total tumor volume to predict outcome after radiotherapy 71.

Although EMH expression has been well described in HNSCC in current literature, there are still opportunities for future studies. In many publications, EMH expression was studied using tissue microarrays (TMAs) 72. In a TMA, tumor tissues from multiple patients are placed on a single histological slide 73. This technique allows for high throughput in determining biomarker expression in patients, but also introduces the risk of sampling bias. Unfortunately, EMH expression may vary widely within tumors, as hypoxia may occur more often in cells that are located distantly from microvessels. This will result in intratumor heterogeneity in the expression of EMH. Positive staining for hypoxic markers is often found in areas of necrosis (perinecrotic staining patterns), which is by some considered proof of “actual hypoxia.” Alternatively, diffuse expression of HIF-1a may also be observed, and is thought to derive from an oncogene-driven overexpression or stabilization of HIF-1a. These expression patterns may be visualized better in whole-slide tissue sections, rather than TMAs. Unfortunately, staining patterns in whole slides have only been described in two studies. Perinecrotic CA-IX staining was associated with worse outcome in a large cohort of LSCC patients treated with ARCON 24. In fact, staining pattern was a stronger predictor of outcome than the percentage of positive staining cells. In a smaller study of OPSCC patients, there was no difference between perinecrotic and diffuse HIF-1a staining patterns 20.

In the past years, HPV+ HNSCC has emerged as a separate entity with a difference in tumor biology, but also a better response to therapy. There may be differences in the prevalence of tumor hypoxia in HPV+ cancers and tumor hypoxia might also affect the prognosis of HPV+ cancers differently than HPV− cancers. This should be investigated in future studies. Finally, the effect of therapies that focus on hypoxia, either through improvement of tumor oxygenation, or by targeting hypoxic tumor cells should be the subject of future studies. Promising therapies include ARCON, the combination of radiotherapy with carbogen gas breathing and nicotinamide administration, which is currently tested in phase III trials 10,74. Delivering increased radiotherapy doses to hypoxic areas in a tumor by IMRT “dose-painting” is also considered, but not yet widely applied 75,76. Alternatively, the hypoxia-sensitizer nimorazole may be added to primary radiotherapy 11. The efficacy of nimorazole administration in HNSCC has been shown in trials within the DAHANCA group 77. Finally, surgery may also be a treatment option for hypoxic tumors, to decrease the hypoxic or therapy-resistant fraction. While devascularization of the surgical field may lead to hypoxia in the direct postoperative phase, revascularization occurs as early as several days after surgery 78. This may increase oxygenation, making remaining tumor cells more susceptible to postoperative radiotherapy. Future studies should investigate the feasibility of such a multimodal approach and the effect on survival of patients with hypoxic tumors.

Several limitations of this review and the identified literature should be considered. In the literature, many ways to score biomarker positivity were used. Most studies scored percentages of positive staining cells, while others also took into account staining intensity, or combined the two using a H-score. If HIF-1a expression will be used in treatment selection, a validated, and reproducible scoring strategy should be employed, preferably without software imaging analysis, that may not be available in all centers. Moreover, the different cutoff points, as well as large heterogeneity in terms of tumor subsite and tumor stages did not allow for proper meta-analysis of the extracted data. As the cutoff points used in the individual studies were most often the ideal cutoff values for each individual data set, data pooling may introduce bias, and give an overestimation of the effect. Therefore, we have not performed this, in contrast to an earlier review on HIF-1a only 79. To provide a visual overview of the results, a forest plot is provided.

Another limitation of this review is that we did not include hypoxia gene expression profiles in the search. Several articles describe such profiles in head and neck cancer 80–84. In the present study we have chosen to focus on IHC, as HNSCC is still highly prevalent in resource-limited areas. Compared to techniques like quantitative PCR (qPCR) or the use of microarrays, IHC may be performed at relatively low cost.

## Conclusion

In this systematic review, we identified HIF-1a, HIF-2a, CA-IX, GLUT-1, and OPN as the best studied endogenous markers of tumor hypoxia. In general, expression of these biomarkers was associated with worse survival, almost regardless of the therapy provided. These proteins are not only biomarkers, but are also part of cellular survival mechanisms. Therefore, EMH overexpression may result in worse prognosis not only due to hypoxia, but also because of a more aggressive tumor phenotype. The effect of tumor hypoxia in HNSCC patients warrants further investigation. Studies should investigate the best treatment option for hypoxic tumors, for instance hypoxia-modified radiotherapy schedules, targeted therapies against hypoxic cells or excision of the hypoxic tissue to improve radiation sensitivity. Knowledge on the tumor hypoxia status will help clinicians to select tailored treatments for each individual patient and thus enable personalized cancer care.

## Conflict of Interest

None declared.

## Supporting Information

**Table S1.**Search strategy.

**Table S2.** Critical appraisal of the excluded studies.

**Table S3.** PRISMA checklist.
