# Supplementary material for: Clinical implications of hypoxia biomarker expression in head and neck squamous cell carcinoma: a systematic review
Source: Cancer Med. 2015 Apr 27;4(7):1101–16. doi: 10.1002/cam4.460 (PMC4529348; doi:10.1002/cam4.460)
Supplement: Supplementary file 1 [file cam40004-1101-sd1.docx]

## Table S1. Search strategy

| **PubMed** | | |
| --- | --- | --- |
| **#1** | (("upper aerodigestive tract"[tiab] OR UADT[tiab] OR (Head[tiab] OR Neck[tiab]OR “Head and neck”[Tiab] OR oropharyn*[Tiab] OR pharyn*[Tiab] OR tonsil*[Tiab] OR “alveolar process”[Tiab] OR Palate[tiab] OR oral[Tiab] OR tong*[Tiab] OR laryn*[Tiab] OR mouth[tiab] OR nasopharyn*[Tiab] OR squamous[tiab] ) AND (Cance*[tiab] OR neoplasm*[tiab] OR malignan*[tiab] OR Tumo*[tiab] OR Tumou*[tiab] OR Carcinom*[tiab] OR oncolog*[tiab] OR adenocarci*[tiab])) OR “Head and Neck Neoplasms”[MeSH] OR “Carcinoma, squamous cell of head and neck”[Supplementary Concept] | *Domain* |
| **#2** | hypox*[tiab] OR HIF-1*[tiab] OR HIF1*[tiab] OR HIF-2*[tiab] OR “HIF2”[tiab] OR Oxygena*[tiab] OR “carbonic anhydrase-9” [tiab] OR “CA-9” [tiab] OR “CA-IX”[tiab] OR “carbonic anhydrase”[tiab] OR Osteopontin[tiab] OR Furin[tiab] OR “Hypoxia-Inducible Factor 1”[MeSH] OR “Cell Hypoxia”[MeSH] OR HIF1[tiab] OR MOP1[tiab] OR  PASD8[tiab] OR HIF-1A[tiab] OR bHLHe78[tiab] OR HIF-1alpha[tiab] OR HIF1-ALPHA[tiab] OR “hypoxia-inducible factor 1-alpha”[tiab] OR HIF-1-alpha[tiab] OR “member of PAS protein 1”[tiab] OR “ARNT interacting protein”[tiab] OR “ARNT-interacting protein”[tiab] OR “member of PAS superfamily 1”[tiab] OR “hypoxia-inducible factor1alpha”[tiab] OR “PAS domain-containing protein 8”[tiab] OR “basic-helix-loop-helix-PAS protein MOP1”[tiab] OR “class E basic helix-loop-helix protein 78”[tiab] OR “hypoxia-inducible factor 1 alpha isoform I.3”[tiab] OR “hypoxia-inducible factor 1, alpha subunit (basic helix-loop-helix transcription factor) ”[tiab] OR “carbonic anhydrase 9”[tiab] OR pMW1[tiab] OR CA-IX[tiab] OR P54/58N[tiab] OR “membrane antigen MN”[tiab] OR “carbonic dehydratase”[tiab] OR “carbonate dehydratase IX”[tiab] OR “RCC-associated antigen G250”[tiab] OR “RCC-associated protein G250”[tiab] OR “renal cell carcinoma-associated antigen G250”[tiab] OR MN[tiab] OR CAIX[tiab] OR OPN[tiab] OR BNSP[tiab] OR BSPI[tiab] OR ETA-1[tiab] OR osteopontin[tiab] OR uropontin[tiab] OR nephropontin[tiab] OR“SPP1/CALPHA1 fusion”[tiab] OR  “urinary stone protein”[tiab] OR “early T-lymphocyte activation 1”[tiab] OR “immunoglobulin alpha 1 heavy chain constant region fusion protein”[tiab] OR “ecreted phosphoprotein 1”[tiab] | *Determinant* |
| **#3** | #1 AND #2 | *Final search* |
| **EMBASE** | | |
| **#1** | (hypox*:ab,ti OR oxygena*:ab,ti OR 'HIF':ab,ti OR HIF*:ab,ti OR OR 'hypoxia-inducible factor’:ab,ti OR ‘hypoxia inducible-factor’:ab,ti OR ‘hypoxia inducible factor’:ab,ti :ab,ti 'hypoxia-inducible factor1alpha':ab,ti OR 'carbonic-anhydrase':ab,ti OR 'CA-9':ab,ti OR 'CA-IX':ab,ti OR 'carbonic dehydratase':ab,ti OR 'CAIX':ab,ti OR 'OPN':ab,ti OR 'osteopontin':ab,ti OR 'FUR':ab,ti OR 'furin':ab,ti OR 'cell hypoxia'/exp OR 'oxygenation'/exp OR 'hypoxia inducible factor'/exp) | *Domain* |
| **#2** | (('upper aerodigestive tract' OR 'UADT' OR 'Head' OR 'Neck' OR Oropharyn* OR Pharyn* OR 'Tonsil' OR 'Alveolar process' OR 'palate' OR 'oral' OR Tong* OR Laryn* OR 'mouth' OR Nasopharyn*) AND ('cance' OR neoplasm* OR carcinom* OR oncolog*) OR ‘head and neck cancer'/exp) | *Determinant* |
| **#3** | #1 AND #2 | *Final search* |
