# Supplementary material for: Clinical implications of hypoxia biomarker expression in head and neck squamous cell carcinoma: a systematic review
Source: Cancer Med. 2015 Apr 27;4(7):1101–16. doi: 10.1002/cam4.460 (PMC4529348; doi:10.1002/cam4.460)
Supplement: Supplementary file 2 [file cam40004-1101-sd2.docx]

## Table S2. Critical appraisal of the excluded studies

| Study | SP | SA | PF | O | C | AR | B |
| --- | --- | --- | --- | --- | --- | --- | --- |
| Bache 2006 | H | L | M | L | L | M | 4 |
| Beasley 2002 | H | H | M | L | M | L | 4 |
| Chan 2007 | H | H | L | L | M | M | 4 |
| Chien 2012 | H | L | M | L | M | M | 5 |
| Choi 2007 | H | L | M | L | M | M | 5 |
| De Schutter 2005 | H | L | M | L | M | L | 4 |
| Eckert 2010 | H | L | L | L | M | M | 4 |
| Etiz 2013 | H | H | L | L | M | M | 4 |
| Hoogsteen 2005 | M | H | M | L | M | M | 4 |
| Kappler 2008 | H | H | M | L | H | H | 7 |
| Klimowicz 2013 | M | H | M | L | H | L | 4 |
| Kondo 2011 | H | H | L | L | H | M | 5 |
| Kong 2009 | M | H | L | L | M | H | 4 |
| Koukourakis 2008 | H | L | L | L | H | M | 5 |
| Koukourakis 2002 | H | L | M | L | M | L | 4 |
| Koukourakis 2001 | H | L | L | L | M | H | 5 |
| Li 2012 | H | H | M | L | H | H | 7 |
| Lin 2008 | H | H | M | L | M | L | 4 |
| Liu 2008 | H | H | L | L | H | H | 6 |
| Oliver 2004 | H | L | M | L | H | M | 6 |
| Shou 2012 | H | L | M | L | M | L | 4 |
| Uehara 2009 | H | H | L | L | H | H | 6 |

SP: study participation (Consecutive cohort? Adequately described for T-stage, N-stage and treatment?); SA: study attrition (Were all patients confirmed alive/deceased at the end of follow-up?); PF: prognostic factor (Is the PF adequately described and determined? Were cut-off values based on the literature? Was the prognostic factor determined the same for all patients?); O: Outcome (Was the outcome well defined?); C: Confounding (were confounders measured and was correction applied if appropriate?); AR: Statistical analysis and reporting (was the used statistical model adequate? Was there selective reporting of results?). B: Bias score according to QUIPS. Low = 0, Moderate = 1, High = 2 points. SA was not included.
